# Satisfaction with chronic obstructive pulmonary disease treatment: results from a multicenter, observational study

**DOI:** 10.1177/1753466619888128

**Published:** 2019-11-24

**Authors:** Marco Contoli, Paola Rogliani, Fabiano Di Marco, Fulvio Braido, Angelo G. Corsico, Christian A. Amici, Roberto Piro, Riccardo Sarzani, Patrizia Lessi, Carla Scognamillo, Nicola Scichilone, Pierachille Santus

**Affiliations:** Department of Medical Sciences, University of Ferrara, Emilia-Romagna, Italy; Department of Experimental Medicine, University of Rome ‘Tor Vergata,’ Rome, Italy; Department of Health Science, Università di Milano, Bergamo, Italy; Department of Internal Medicine, University of Genoa, Genoa, Italy; Division of Respiratory Diseases, IRCCS Policlinico San Matteo Foundation, University of Pavia, Pavia, Italy; Medineos Observational Research, Modena, Emilia-Romagna, Italy; Azienda Unità Sanitaria Locale-IRCCS di Reggio Emilia, Italy; Internal Medicine and Geriatrics, Università Politecnica delle Marche and IRCCS-INRCA, Ancona, Italy; Boehringer Ingelheim Milano, Italy; Boehringer Ingelheim Milano, Italy; DIBIMIS, University of Palermo, Palermo, Italy; Department of Biomedical and Clinical Sciences (DIBIC), Università di Milano, Division of Respiratory Diseases, ‘L. Sacco’ Hospital, Via G.B. Grassi 74, Milan 20157, Italy

**Keywords:** adherence, COPD, treatment satisfaction

## Abstract

**Background:**

Understanding the level of patients’ satisfaction with treatment and its determinants have the potential to impact therapeutic management and clinical outcome in chronic conditions such as chronic obstructive pulmonary disease (COPD).

**Methods:**

A national, multicenter, longitudinal, observational study of COPD from 20 Italian pulmonary centers to explore patients’ satisfaction to treatment [assessed by the Treatment Satisfaction Questionnaire, 9 items (TSQM-9)] and association with clinical parameters [including dyspnea score, COPD Assessment Test (CAT) score, exacerbation rate], adherence to treatment [Morisky Medication-Taking Adherence Scale (MMAS-4)], illness perception [evaluated by Brief Illness Perception Questionnaire (B-IPQ)] in a 1-year follow up.

**Results:**

A total of 401 COPD patients were enrolled [69.4% group B Global Initiative for COPD (GOLD), considering 366 patients with available GOLD 2017 classification at enrollment]. At enrollment, satisfaction with treatment was moderate, being TSQM-9 mean scores for effectiveness 64.2 [95% confidence interval (CI) 62.5–65.9], for convenience 75.8 (95% CI 74.2–77.3), and for global satisfaction 65.7 (95% CI 64.0–67.4). Global satisfaction was negatively associated with disease perception (β = −0.4709, *p* < 0.0001), and grade of dyspnea (β = −4.2564, *p* = 0.009). Satisfaction with treatment was lower in patients with poor compared with optimal adherence to treatment (β = −4.5608, *p* = 0.002). Changes in inhalation regimens during follow up did not modify the satisfaction with treatment.

**Conclusions:**

The results of this real-life study showed that the patients’ satisfaction with treatments is only moderate in COPD. A high grade of patients’ satisfaction is associated mainly with a low perception of the disease, high adherence to treatment and lower level of dyspnea.

**Trial Registration:**

Clinicaltrials.gov identifier: NCT02689492

*The reviews of this paper are available via the supplemental material section.*

## Introduction

Chronic obstructive pulmonary disease (COPD) is characterized by persistent respiratory symptoms, exercise intolerance, and airflow limitation due to airway or alveolar abnormalities, commonly caused by a substantial exposure to noxious particles or gases, cigarette smoking being the most relevant risk factor.^1^ COPD is one of the most common causes of morbidity, mortality, and increased health costs among chronic diseases.^2^

The success of any therapy for chronic conditions is determined by the adherence to long-term therapy, defined by the World Health Organization (WHO) as ‘the extent to which a person’s behavior (taking medication, following a diet, or executing lifestyle changes) corresponds with the agreed recommendations from a healthcare provider.’^3^ As this concept of adherence is expressed, it includes not only compliance to pharmacological and nonpharmacological treatments but also the extent to which the patient’s behavior matches agreed recommendations from the prescriber (e.g. smoking cessation, dietary restriction, regular physical activities, periodical medical consultations). Long-term adherence is a major unmet medical need in chronic conditions, negatively influencing short- and long-term prognosis. In addition, poor adherence to treatment increases disease-related costs and may contribute to treatment gaps in COPD care.^3,4^ Almost half of patients with COPD do not adhere to their medications.^5^

Patient satisfaction with their medications is shown to affect treatment-related factors, such as their likelihood of continuing use of their medication, using their medication correctly, and adherence of their medication regimen.^6,7^

Limited information is available on the long-term treatment satisfaction and potential correlation with treatment adherence of patients with COPD in the real-life setting, as well as on the impact of satisfaction on clinical outcomes. To address this gap of information, we performed a national multicenter longitudinal observational study to primarily explore the patients’ satisfaction with COPD medical treatment in a clinical, real-world setting. Furthermore, we evaluated if and how this is related to clinical parameters, quality of life, illness perception and treatment adherence evolution, during a 12-month follow up.

Understanding the impact of treatment patient satisfaction on clinical outcomes could help identify determinants of poor adherence and highlight potential actions to improve success of COPD management.

## Methods

### Study design and population

The SATisfaction and adherence to COPD treatment (SAT) study was a multicenter, non-interventional (observational) cohort study. A detailed description of the study design and procedures are available in Supplementary Appendix 1. Briefly, consecutive COPD patients were enrolled between November 2015 and September 2016. Patients were followed up for 1 year, with an intermediate evaluation after 6 (±1) months from baseline. The study conformed to the Declaration of Helsinki. The local institutional ethics committees approved the work, and informed written consent was obtained from each participant. The study is registered [ClinicalTrials.gov identifier: NCT02689492].

All patients were aged > 40 years and had COPD according to symptoms, spirometry results, and the standard definition according to the Global Initiative for COPD (GOLD). Patients had to be free from a COPD exacerbation since at least 3 months and on stable, inhaled treatment for at least 3 months. The main exclusion criteria included patients naïve/without chronic, inhaled treatment, concomitant diagnosis of asthma.

### Study procedures, variables, and outcomes

The assessment and the treatment of the enrolled patients were applied according to standard clinical practice. No treatment was administered to the patients on the protocol basis. At baseline, socio-demographic variables, smoking habits, medical history, lung function test (by means of spirometry), and history of COPD exacerbations in the previous year were collected (Table 1). The exacerbation of COPD was defined as a symptomatic deterioration requiring treatment with antibiotic agents, systemic corticosteroids (moderate), hospitalization, or a combination of these (severe).^8^ At each visit, data on switching/modification of inhaled treatments and exacerbation events occurring from the previous visit were collected. Furthermore, at each study visit, the physicians were asked to collect by specific and validated questionnaires: (a) patients’ satisfaction with COPD medical treatments evaluated through the Treatment Satisfaction Questionnaire, 9 items (TSQM-9; ranging between 0 and 100, the higher the score, the higher the grade of satisfaction);^9,10^ (b) patient disease perception, evaluated by means of the Brief Illness Perception Questionnaire (B-IPQ; ranging between 0 and 80, the higher scores indicate a more threatening notion of COPD by the patient);^11^ (c) adherence to COPD treatment, evaluated by means of the Morisky Medication-Taking Adherence Scale (MMAS-4; ranging between 0 and 4, the higher scores indicate greater adherence to therapy);^12^ (d) disease-related health status by the COPD Assessment Test (CAT; CAT total score of ⩾10 is used by in the GOLD document 1 to classify COPD patients as highly symptomatic);^13^ and (e) dyspnea severity by the Modified Medical Research Council (mMRC; ranging between 0 and 4, mMRC score of ⩾2 is used by in the GOLD document (1) to classify COPD patients as highly symptomatic) scale.^14^ No minimal clinically important difference has been validated for the TSQM-9, B-IPQ and MMAS-4 questionnaires. The most reliable estimate of the minimum clinically important difference of the CAT questionnaire is 2 points.^15^ A difference in one unit between two consecutive measurements in the mMRC score has been proposed as clinically meaningful in COPD.^16^ A detailed description of the composition of the questionnaires and scoring interpretation is available in Supplementary Appendix 1.

**Table 1.** Assessment schedule of the study.

		Visit 1	Visit 2	Visit 3
		Baseline	Follow-up 1	Follow-up 2
Assessment	Months:	0	6 (±1)	12 (±1)
**Eligibility criteria** Inclusion and exclusion criteria, informed consent, and privacy form		X		
**Baseline information** Socio-demographic variables: age, sex, race, geographic location, housing situation, marital status, educational and employment status		X		
**Weight, body mass index**		X	X	X
**Physical examination**		X	X	X
**Life habits** Smoking (yes/no, number of pack/years, smoking duration)		X	X	X
**Medical history and concomitant diseases** Charlson Comorbidity Index		X	X	X
**COPD medical history:** Date of COPD diagnosis (years from diagnosis); number of COPD exacerbations/year during the previous year to enrollment visit		X		
**Functional assessment** Lung function test results (FEV1, FVC, FEV1% of the predicted, RV, TLC, DLCO) according to clinical practice		X	X	X
**CAT questionnaire**		X	X	X
COPD exacerbations^*^ **after enrollment** Onset and resolution date Severity (mild, moderate, severe)		X	X	X
**Disease severity (GOLD 2017 guidelines)**		X	X	X
**Medications related to COPD, COPD exacerbations and adverse events** (LABA, LAMA, SABA, SAMA, ICS/LABA, steroids, antibiotics, etc.): drug, dose, frequency, duration of therapy Long-term oxygen therapy (liquid or concentrate)		X	X	X
Change of therapy during observation period and reason for change.			X	X
**Nonpharmacological treatment** Pulmonary rehabilitation		X	X	X
**Patient-reported outcome questionnaires/scales**				
TSQM-9		X	X	X
mMRC		X	X	X
CAT		X	X	X
MMAS-4		X	X	X
Brief Illness Perception Questionnaire		X	X	X
Awareness structured interview		X	X	X
**Serious adverse events assessment**			X	X

* An exacerbation was defined as an increase or new onset of more than one symptom (cough, sputum, wheezing, dyspnea or chest tightness) with at least one symptom lasting at least 3 days and leading to the patient’s attending physician to initiate treatment with systemic steroids, antibiotics (moderate exacerbation), or hospital admission (severe exacerbation).

CAT, COPD Assessment Test; COPD, chronic obstructive pulmonary disease; DLCO, diffusing capacity of the lungs for carbon monoxide; FEV1, forced expiratory volume at 1 s; FVC, forced vital capacity; GOLD, Global Initiative for COPD; ICS, inhaled corticosteroid; LABA, long-acting beta agonist; LAMA, long-acting muscarinic antagonist; MMAS-4, Morisky Medication-Taking Adherence Scale; mMRC, Modified Medical Research Council Scale; RV, residual volume; SABA, short-acting beta agonist; SAMA, short-acting muscarinic antagonist; TLC, total lung capacity; TSQM-9, Treatment Satisfaction Questionnaire, 9 items.

The primary endpoint was to describe patients’ satisfaction with COPD medical treatments by means of the TSQM-9 during a 12-month observation period in a real-world setting The secondary endpoints included: (a) disease perception, adherence to treatment, health status, and dyspnea over the 12-month observation period; and (b) identification of factors associated with patient satisfaction with COPD medical treatments.

### Sample size and statistical analyses

Since this was a descriptive study, no formal statistical hypotheses were set. The sample size was determined based on feasibility: according to the number of patients managed by the centers involved in the study, the inclusion of 400 participants fulfilling the inclusion/exclusion criteria was deemed feasible in the defined enrollment period. Assuming an overall dropout rate ranging from 5% to 20%, the foreseen total number of evaluable patients ranged from between 380 and 320, respectively. Under these assumptions, the achievable precision for the estimates of the primary endpoint, namely, the mean TSQM-9 global satisfaction score, was evaluated, considering previous surveys with TSQM on patients with other chronic conditions^17,18^ which showed a mean ± standard deviation (SD) score accounting for 54.4 ± 21.4 and 79.7 ± 16.6 points, respectively. In all considered scenarios, the achievable precision was deemed adequate, since the half-widths of the 95% confidence intervals (CIs) of the mean^19^ were always *<*2.5 points, and the relative errors of the estimates were always *<*30%.

Descriptive statistics were used to evaluate socio-demographic and clinical variables at the baseline visit and during the study period. Repeated-measures linear regression models were used^20^ to estimate the β coefficients for the evaluation of the associations between the three TSQM-9 treatment satisfaction domain scores (effectiveness, convenience, and global satisfaction of COPD treatments, each ranging 0–100 points) and the following independent factors: age, sex, B-IPQ total score, forced expiratory volume at 1 s (FEV1) percentage of predicted, number of annual exacerbations, MMAS-4 score (poor/suboptimal *versus* optimal), mMRC dyspnea grade, and COPD therapeutic regimen. Only patients with data available both at enrollment and at 12-month follow-up visits were included in the regression models.

The patients switching or stopping treatment during the observation period were not withdrawn from the study. Patients with missing values at 6-month follow-up visit were not excluded for the primary outcome analysis. Database management and data analysis were performed using SAS® 9.4.

## Results

The study population consisted of 401 patients enrolled in a 10-month period. At baseline, considering the 366 patients with available retrospective data, 38.5% of patients reported having experienced at least one exacerbation in the year prior to study and the clear majority of patients belonged to GOLD group B,^21^ characterized by relatively few exacerbations but significant disease-related impairment of health status and symptom severity (Table 2). Inhaled treatments are reported in Table 2. The average number of exacerbations per patient during observation period was 0.3 event/patient (SD = 0.6) and 99 patients (24.7%) had at least one new exacerbation during the 12-month observation period. Only 9 events (2.2 events/100 patients) of hospitalization occurred during the follow-up period.

**Table 2.** Demographics and clinical characteristics of the patients at enrollment.

Age (mean ± SD)	71.7 ± 7.6
**Sex**	
Male	299 (74.6%)
Female	102 (25.4%)
**COPD**	
Duration, years (median, 25th–75th percentile)	4.9 (2.1–9.2)
Age at diagnosis, years, mean ± SD	65.3 ± 9.0
**Patients with comorbidities**	339 (84.5%)
**GOLD 2017 group classification (*n* = 366)**	
Group A	70 (19.1%)
Group B	254 (69.4%)
Group C	2 (0.5%)
Group D	40 (11.0%)
Unknown	35
**Exacerbations during last year (*n* = 366)**	
None	225 (61.5%)
⩾1	141 (38.5%)
Unknown	35
**Treatment for COPD at enrollment** ^*^	
LABA+LAMA+ICS	153 (38.2%)
LABA+LAMA	99 (24.7%)
LAMA	92 (22.9%)
SABA or SAMA on demand	43 (10.7%)
LABA+ICS	36 (9.0%)
LABA	18 (4.5%)
ICS	4 (1.0%)
Other treatments for COPD	16 (4.0%)

* A patient could have received more than one treatment. The following treatment categories are mutually exclusive: LAMA, LABA, ICS, LABA+LAMA, LABA+ICS, LABA+LAMA+ICS.

COPD, chronic obstructive pulmonary disease; GOLD, Global Initiative for COPD; ICS, inhaled corticosteroid; LABA, long-acting beta agonist; LAMA, long-acting muscarinic antagonist; SABA, short-acting beta agonist; SAMA, short-acting muscarinic antagonist; SD, standard deviation.

### Patient satisfaction with COPD medical treatments

**Figure 1.**
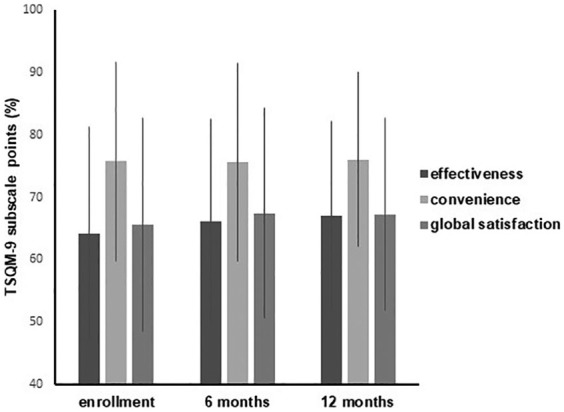
Percentage scores related to effectiveness, convenience, and global satisfaction domains of the TSQM-9 assessments at enrollment, at the 6-month, and 12-month visits. No clinically relevant difference is evident in the TSQM-9 scores during the study period. The satisfaction to treatment in these patients is moderate. The bar represents the standard deviation. TSQM-9, Treatment Satisfaction Questionnaire, 9 items.

The level of patient satisfaction with treatment for COPD (primary endpoint) was evaluated at baseline and during the study period according to the scores recorded for the three TSQM-9 domains (effectiveness, convenience, and global satisfaction). The results of TSQM-9 at baseline were available for 390 patients, while the TSQM-9 assessments at the 6-month and 12-month visits were available for 355 patients and 304 patients, respectively. Overall, no change was found in the TSQM-9 scores during the study period (Figure 1). Overall, the satisfaction scores, and particularly those related to effectiveness and global satisfaction domains, classified the satisfaction with treatment in these patients as moderate.

### Disease perception

Patient perception of the disease was evaluated using the B-IPQ tool. The change in perception of the disease along the study was evaluated against the baseline visit in patients with scores available at enrollment, at the 6-month, and 12-month visits. Overall, no change in disease perception was recorded over the 12-month observation (Table 3).

**Table 3.** Assessment of disease perception, treatment adherence, health status, and dyspnea over the 12-month study period.

Domain	Score at enrollment ± SD	Variation over the study period	
		6-month visit^*^ ± SD	12-month visit^**^ ± SD
Perception (B-IPQ total score)	41.8 ± 11.3 (*n* = 396) 95% CI 40.7–42.9	0.5 ± 7.6 (*n* = 348) 95% CI −0.3 to 1.3	1.3 ± 9.3 (*n* = 300) 95% CI 0.2–2.3
Adherence (MMAS-4 total score)	3.4 ± 0.9 (*n* = 401) 95% CI 3.3–3.5	0.1 ± 0.8 (*n* = 360) 95% CI 0.0–0.1	0.1 ± 0.9 (*n* = 308) 95% CI 0.0–0.2
Health status (CAT total score)	15.7 ± 7.8 (*n* = 401) 95% CI 14.9–16.5	0.4 ± 5.9 (*n* = 360) 95% CI −0.2 to 1.0	0.0 ± 6.8 (*n* = 307) 95% CI −0.7 to 0.8
Dyspnea (mMRC total score)	1.6 ± 1.1 (*n* = 400) 95% CI 1.5–1.8	0.1 ± 0.8 (*n* = 358) 95% CI 0.1–0.2	0.1 ± 0.9 (*n* = 305) 95% CI 0.0–0.2

* In this analysis, only evaluable patients at 6 months with score available both at enrollment and 6-month follow-up visits were considered.

** In this analysis, only evaluable patients at 12 months with score available both at enrollment and 12-month follow-up visits were considered.

B-IPQ, Brief Illness Perception Questionnaire; CAT, COPD Assessment Test; CI, confidence interval; COPD, chronic obstructive pulmonary disease; MMAS, Morisky Medication-Taking Adherence Scale; mMRC, Modified Medical Research Council Scale; SD, standard deviation.

### Adherence to treatment for COPD

Adherence to treatment was measured using the MMAS-4 questionnaire. The mean MMAS-4 total score at enrollment was 3.4 ± 0.9 and remained substantially constant along the study period. The intrasubject variation of MMAS-4 scoring was also minimal, resulting in 0.1 points both at 6-month and 12-month visits (95% CI 0.0–0.1 and 95% CI 0.0–0.2, respectively). No change in adherence to treatment was observed over the study period (Table 3).

### Health status, and dyspnea score

Health status of the patients was evaluated by CAT. At enrollment visit, most of the patients (49.6%) scored between 10 and 20 points. Overall, the mean CAT score did not change over 12 months. (Table 3). In 126 patients (41%) we found an increase at 12 months compared with baseline of CAT ⩾ 2 points (minimal clinically important difference) while in 124 patients (40%), a decrease ⩾ 2 points (Supplementary Appendix 1, Table 1). Dyspnea was evaluated by the mMRC questionnaire. At baseline, mean mMRC questionnaire score was 1.6 ± 1.1 and did not substantially differ over the 12-month study period. Overall, no relevant variations in dyspnea grade were observed over the 12-month observational study (Table 3). In 67 patients (22%), we found an increase at 12 months compared with baseline of mMRC scale ⩾ 1 point (minimal clinically important difference), while in 78 patients (26%) a decrease ⩾ 1 point (Supplementary Appendix 1, Table 1).

### Factors associated with treatment satisfaction

In patients with a decreased CAT score (reflecting the improvement of the impact of COPD on a patient’s life), we found a numerical increase of all the domains of the TSQM-9 questionnaire (meaning an improvement of patients’ satisfaction with treatment). Similarly, we found an increase of all the domains of the TSQM-9 questionnaire in COPD patients with a decrease of at least 1 point in the mMRC scale at the end of the study period compared with baseline (Supplementary Appendix 1, Table 1). Overall, no statistically significant associations were found between patient satisfaction (any of the TSQM-9 domains) and the frequency of exacerbations occurring during the study period. A weak statistically significant negative correlation was found between the convenience item of the patient satisfaction score and the total number of hospitalizations (ρ = −0.13, *p* value = 0.02).

A multivariate linear regression analysis model was implemented to evaluate the relationship between the demographic data, clinical parameters, or patient-reported outcomes and the patients’ satisfaction with COPD medical treatments. No statistically significant associations were found between any of the TSQM-9 domains and demographics (age and sex), FEV1 values, and number of exacerbations. Similarly, the COPD treatment modalities [inhaled corticosteroid (ICS) treatment *versus* any non-ICS treatment] was not significantly associated with patients’ satisfaction with treatment. Interestingly, we found that the only clinical variable significantly associated with patient satisfaction with medical treatments was the dyspnea score. The global satisfaction domain of the TSQM-9 score was negatively correlated with the mMRC (β = 4.2564, *p* = 0.009); the patients with a higher grade of dyspnea (mMRC ⩾ 2) had a mean global satisfaction score of 4.3 points lower than patients with a lower grade of dyspnea (mMRC < 2; Table 4).

**Table 4.** Multivariate linear regression analysis on TSQM-9 domains compared to demographic data, clinical parameters, and patient-reported outcomes.

	Effectiveness		Convenience		Global satisfaction	
	β ± SE	*p*	β ± SE	*p*	β ± SE	*p*
Intercept	85.06 ± 8.18	**<0.0001**	106.32 ± 7.70	**<0.0001**	93.55 ± 8.09	**<0.0001**
Visit (12 months *versus* enrollment)	3.27 ± 1.43	**0.02**	1.51 ± 1.19	0.20	2.57 ± 1.27	**0.04**
Age	−0.02 ± 0.10	0.87	−0.18 ± 0.09	0.06	−0.06 ± 0.10	0.57
Sex (female *versus* male)	0.71 ± 1.71	0.68	−1.29 ± 1.59	0.42	0.75 ± 1.69	0.66
FEV1 (%) (⩾50% *versus* <50%)	0.24 ± 1.72	0.89	−0.71 ± 1.56	0.65	−1.25 ± 1.67	0.45
Annual exacerbations, *n* (⩾1 *versus* none)	1.17 ± 1.50	0.44	−0.95 ± 1.32	0.47	1.07 ± 1.40	0.44
Dyspnea (mMRC ⩾ 2 *versus* <2)	−3.15 ± 1.69	0.06	−0.26 ± 1.52	0.86	−4.26 ± 1.61	**<0.01**
COPD treatment (without ICS *versus* with ICS)	−0.85 ± 1.48	0.56	0.54 ± 1.37	0.69	−0.82 ± 1.45	0.57
Disease perception (B-IPQ)	−0.41 ± 0.08	**<0.0001**	−0.34 ± 0.07	**<0.0001**	−0.47 ± 0.07	**<0.0001**
Adherence (MMAS-4 poor/suboptimal *versus* optimal)	−2.05 ± 1.55	0.19	−6.62 ± 1.40	**<0.0001**	−4.56 ± 1.49	**<0.01**

Poor/suboptimal adherence class corresponds to MMAS-4 score = 0–3; optimal adherence class corresponds to MMAS-4 score = 4. Statistically significant values are in bold.

B-IPQ, Brief Illness Perception Questionnaire; β, regression coefficient; COPD, chronic obstructive pulmonary disease; FEV1, forced expiratory volume after 1 s; ICS, inhaled corticosteroid; MMAS, Morisky Medication-Taking Adherence Scale; mMRC, Modified Medical Research Council Scale; SE, standard error; TSQM-9, Treatment Satisfaction Questionnaire, 9 items.

Notably, a significant negative association (β = 0.4709, *p* < 0.0001) emerged between the scores to all items of the TSQM-9 questionnaire recorded at the end of the study and the disease perception (B-IPQ) score (Table 4). COPD patients with poor/suboptimal adherence to treatment (MMAS-4 scores 0–3) had a lower mean convenience score (about 6.6 points less; *p* < 0.0001) and lower global satisfaction score (of about 4.6 points; β = 4,5608, *p* < 0.002) compared with patients with optimal adherence (MMAS-4 score = 4). In the multivariate linear regression low/suboptimal adherence to treatment was associated with low satisfaction to treatment (Table 4).

The levels of satisfaction to treatments were evaluated in the COPD patients according to the inhaled treatment regimens ongoing at the end of the study. Similar mean scores for the three domains of TSQM-9 were found irrespective of the treatment modality (mono, dual, or triple therapy; Table 5). Similar levels of TSQM-9 scores were found during the 12-month follow-up period between patients who changed (switchers, *n* = 73) and who did not change (nonswitchers, *n* = 231) their pharmacological inhalatory regimens.

**Table 5.** Treatment satisfaction in patients stratified according to the type of ongoing COPD treatment at the 12-month follow-up visit.

	COPD treatment classes							
	ICS	LABA	LABA ICS	LABA LAMA	LABA LAMA ICS	LAMA	SABA or SAMA on demand	Other
**TSQM-9 domains**								
Patients, *n*	2	14	26	86	110	63	39	24
Effectiveness score	75.0 ± 11.8	68.7 ± 15.3	70.7 ± 12.2	67.0 ± 15.3	66.1 ± 15.5	67.7 ± 13.5	69.4 ± 13.0	65.0 ± 15.4
Convenience score	69.4 ± 3.9	78.6 ± 14.6	79.3 ± 13.3	78.0 ± 14.5	74.2 ± 13.1	75.3 ± 14.8	77.4 ± 14.8	72.9 ± 13.7
Global satisfaction score	60.7 ± 15.2	69.9 ± 12.6	68.7 ± 14.9	67.7 ± 15.4	66.7 ± 14.6	68.0 ± 14.3	67.0 ± 12.9	66.4 ± 13.7

Only patients with available TSQM-9 domain scores and at least one COPD treatment at the 12-month follow-up visit were considered. A patient may have received more than one treatment. Mean ± SD values are indicated for the TSQM-9 scores.

COPD, chronic obstructive pulmonary disease; ICS, inhaled corticosteroid; LABA, long-acting beta agonist; LAMA, long-acting muscarinic antagonist; SABA, short-acting beta agonist; SAMA, short-acting muscarinic antagonist; SD, standard deviation; TSQM-9, Treatment Satisfaction Questionnaire, 9 items.

## Discussion

Previous surveys have been conducted in cohorts of patients with COPD to explore perception or awareness of disease severity,^22–24^ self-management and improvement of quality of life,^25^ and adherence to therapies.^9,11^ However, this national, multicenter study is the first comprehensive analysis that corelates different domains of patient satisfaction with therapy for COPD with adherence, health status, and illness perception, by using five different validated questionnaires and clinical parameters during a 12-month follow up in a clinical real-world setting. Overall, we found that patient satisfaction could be considerate, according to the questionnaire adopted, or only moderate. Interestingly, patient satisfaction was associated with a low perception of the disease and high adherence to treatment. Dyspnea score was the only clinical parameter found to be negatively associated with patient satisfaction with treatment. A weak statistically significant negative correlation between the convenience item of the patients’ satisfaction score (exploring satisfaction to treatment regimen, dosing complexity, and frequency) and the total number of hospitalizations was found. This finding suggests a greater satisfaction in participants who had less COPD-related hospitalizations. However, it should be noted that the magnitude of these correlations is very limited, meaning that they may not actually be clinically relevant.

The demographics and the clinical features of the patients enrolled in this real-world study (mainly group B with low rate/no exacerbation in the previous year) were representative and in line with the general population of COPD^26^ included in several other real-life observational studies.^9,22,23,27^

In our study, we found that the satisfaction of patients with ongoing therapy after 1 year was only moderate and did not vary significantly over the 1-year follow up. When disease perception and adherence to treatment were examined over the 12-month period of observation, no statistically significant changes were observed. Similarly, no clinically meaningful variations in health status occurred.

The multivariate regression analysis performed in this study included the clinical parameters related to the most recent grading system for COPD assessment and derived from patient symptoms, namely dyspnea (mMRC score), in addition to the history of exacerbation, adherence, and illness perception.

The results of this analysis revealed that patient satisfaction is associated mainly with a low perception of disease and high adherence to treatment. The negative correlation between all three domains of the satisfaction questionnaire and the B-IPQ score suggests a patient is satisfied when he underestimates his/her disease. Further studies will be required to shed light on this issue.

The association between poor/suboptimal adherence (MMAS-4 score = 0–3) with poor convenience and global satisfaction domains of TSQM-9 clearly supports previous evidence that improving the adherence to treatment can positively influence patient satisfaction with treatments in COPD.^3,17,28^

In our study, patient satisfaction was also influenced by clinical expression of the disease in terms of dyspnea, because of the statistically significant correlation between the global satisfaction domain of TSQM-9 and the mMRC score. Patients who are symptomatic in terms of dyspnea (mMRC score ⩾ 2) are less satisfied by their therapy, irrespective of the ongoing inhalation treatment. This result is of relevance because it may help the clinician to better evaluate and predict patient satisfaction with therapy. Our results demonstrate that dyspnea is the most critical symptom, and this should be considered when selecting the most satisfactory therapy possible, at least for the symptomatic patients with COPD.

The most recent GOLD classification is deemed potentially useful in providing sufficiently strong evidence to prescribe changes of treatment modality (mono, double, triple therapy) according to the disease severity assessed by the GOLD system. However, this real-life analysis on satisfaction and adherence in patients stratified according to the type of ongoing COPD treatment suggests that treatment modality or modification do not impact patient satisfaction (TSQM-9). This information is of relevance to clinicians who should be aware that other determinants over treatment regimens could influence patients’ satisfaction with treatment.

A possible limitation of this study is the relatively low amplitude of the differences between groups or parameters compared in the study, despite being statistically significant. The differences measured in the analysis of TSQM-9 never exceeded 7–10 points, compared with the 0–100-point range of the questionnaire. However, a similar amplitude of significant-point difference has been found in previous reports evaluating satisfaction with treatment.^13,17,18^ Furthermore, the mean scores for patient satisfaction with treatment and adherence are surprisingly high compared with previous studies.^5,11,23^ In this regard, it must be recognized that most of the patients enrolled in the study belong to a cohort of patients already regularly referring to the centers that participated in the study. This bias can influence the two study variables. Finally, considering the descriptive methodological approach used in our study, we recognize that inferential studies are needed to confirm our preliminary findings regarding the putative factors associated with COPD treatment satisfaction.

## Conclusion

The real-life profile of this study may better provide a clear and accurate picture of the daily clinical practice in the management of COPD. In particular, the identification of risk factors associated with poor patient satisfaction with treatment can help suggest novel strategies for improving COPD management.

## Supplemental Material

Author_Response_1 – Supplemental material for Satisfaction with chronic obstructive pulmonary disease treatment: results from a multicenter, observational study Click here for additional data file. Supplemental material, Author_Response_1 for Satisfaction with chronic obstructive pulmonary disease treatment: results from a multicenter, observational study by Marco Contoli, Paola Rogliani, Fabiano Di Marco, Fulvio Braido, Angelo G. Corsico, Christian A. Amici, Roberto Piro, Riccardo Sarzani, Patrizia Lessi, Carla Scognamillo, Nicola Scichilone and Pierachille Santus in Therapeutic Advances in Respiratory Disease

## Supplemental Material

Contoli_et_al_Additional_file20190924 – Supplemental material for Satisfaction with chronic obstructive pulmonary disease treatment: results from a multicenter, observational study Click here for additional data file. Supplemental material, Contoli_et_al_Additional_file20190924 for Satisfaction with chronic obstructive pulmonary disease treatment: results from a multicenter, observational study by Marco Contoli, Paola Rogliani, Fabiano Di Marco, Fulvio Braido, Angelo G. Corsico, Christian A. Amici, Roberto Piro, Riccardo Sarzani, Patrizia Lessi, Carla Scognamillo, Nicola Scichilone and Pierachille Santus in Therapeutic Advances in Respiratory Disease

## Supplemental Material

Reviewer_1_v.1 – Supplemental material for Satisfaction with chronic obstructive pulmonary disease treatment: results from a multicenter, observational study Click here for additional data file. Supplemental material, Reviewer_1_v.1 for Satisfaction with chronic obstructive pulmonary disease treatment: results from a multicenter, observational study by Marco Contoli, Paola Rogliani, Fabiano Di Marco, Fulvio Braido, Angelo G. Corsico, Christian A. Amici, Roberto Piro, Riccardo Sarzani, Patrizia Lessi, Carla Scognamillo, Nicola Scichilone and Pierachille Santus in Therapeutic Advances in Respiratory Disease

## Supplemental Material

Reviewer_2_v.1 – Supplemental material for Satisfaction with chronic obstructive pulmonary disease treatment: results from a multicenter, observational study Click here for additional data file. Supplemental material, Reviewer_2_v.1 for Satisfaction with chronic obstructive pulmonary disease treatment: results from a multicenter, observational study by Marco Contoli, Paola Rogliani, Fabiano Di Marco, Fulvio Braido, Angelo G. Corsico, Christian A. Amici, Roberto Piro, Riccardo Sarzani, Patrizia Lessi, Carla Scognamillo, Nicola Scichilone and Pierachille Santus in Therapeutic Advances in Respiratory Disease
