# Supplementary material for: Satisfaction with chronic obstructive pulmonary disease treatment: results from a multicenter, observational study
Source: Ther Adv Respir Dis. 2019 Nov 24;13:1753466619888128. doi: 10.1177/1753466619888128 (PMC6878607; doi:10.1177/1753466619888128)
Supplement: Contoli_et_al_Additional_file20190924 – Supplemental material for Satisfaction with chronic obstructive pulmonary disease treatment: results from a multicenter, observational study [file Contoli_et_al_Additional_file20190924.pdf]

Patients and methods

Study design.

The SATisfaction and adherence to COPD treatment (SAT; Clinicaltrials.gov ID# NCT02689492) study in patients with COPD was a multi-center, non-interventional (observational) cohort study based mainly on newly collected data.

In 20 Italian Pulmonary Centers, 401 consecutive patients with COPD were enrolled in approximately 10 months. Patients were followed up for 1 year, with an intermediate evaluation after 6 (+/-1) months from baseline, according to current clinical practice in Italy for the management of patients with COPD [1].

No treatment was administered to the patients on the protocol basis since this is a non-interventional study. Assessment and treatment of the enrolled patients were applied according to standard clinical practice. The patients switching or stopping treatment during the observation period were not withdrawn from the study. Patients who stopped treatment during the study were censored at the latest available visit for primary objective evaluation[1].

Setting. All patients, both female and male gender, referring to pulmonary centers were consecutively enrolled according to the following inclusion criteria:

1. Patients aged ≥40 years
2. Patients with a documented diagnosis of COPD
3. Patients with no exacerbations in the last 3 months
4. Patients requiring regular treatment according to guidelines of the Global Strategy for the Diagnosis, Management, and Prevention of Chronic Obstructive Pulmonary Disease (GOLD), i.e.: undergoing stable pharmacological treatment for COPD since at least 3 months

5. Written informed consent to both participation in the study and privacy form
6. Patients capable of discernment and able to read or write in Italian language.

Exclusion criteria were:

1. Patients who are currently participating in a clinical trial on experimental drugs.
2. Patients naïve to pharmacological treatment for COPD
3. Diagnosis of Asthma COPD Overlap Syndrome (ACOS).

The following study exit criteria were established:

1. An individual patient may be withdrawn from the non-interventional study prior to completion if any of the following criteria apply:
2. The patient withdraws consent, with no need to justify his/her decision.
3. The patient is no longer able to participate (according to clinical judgment)
4. Administrative reasons (eg inclusion in a different clinical trial).

Variables. The primary endpoint was patients' satisfaction with COPD medical treatments evaluated through the Treatment Satisfaction Questionnaire, 9 items (TSQM-9) version 1.4 [2, 3] at baseline and at follow-up visits.

The TSQM-9, derived from the original version, has a total of 9 items with responses based on a five-point or seven-point rating scales: effectiveness (3 items), convenience (3 items), and global satisfaction (3 items) [4]. The TSQM-9 domain scores (effectiveness, convenience, and global satisfaction) were calculated as recommended [2, 3]. The scores in each domain ranged from 0 (maximum grade of dissatisfaction) to 100 points (maximum grade of satisfaction).

The secondary endpoints evaluated in this study were:

1) Patient disease perception, evaluated by means of the Brief Illness Perception Questionnaire (B-IPQ), a validated 9-item questionnaire designed to rapidly assess cognitive and emotional

representations of illness [5]. All questionnaire items (except the causal question, item 9) were rated using a 0-to-10 response scale, resulting in a final 0 - 80 score range, being a higher score indicative of a more threatening notion of COPD by the patient. Five of the items assess cognitive illness representations: consequences (Item 1), timeline (Item 2), personal control (Item 3), treatment control (Item 4), and identity (Item 5). Two of the items assess emotional representations: concern (Item 6) and emotions (Item 8). One item assesses illness comprehensibility (Item 7). Assessment of the causal representation is through an opened response item, which asks patients to list the three most important causal factors in their illness (Item 9).

2) Adherence to COPD treatment, evaluated by means of the Morisky Medication Taking Adherence Scale (MMAS-4)[6], a self-reported, medication-taking behavior scale, consisting of four questions about the way patients might experience drug errors or omissions. Each item has a scoring scheme of “Yes” = 0 and “No” = 1. Items were summed to give a non-adherence score ranging from 0 to 4 (42-44), a higher score indicating greater adherence to therapy.

3) Health status, by evaluating two different measures: i) the COPD Assessment Test (CAT) [7] and ii) dyspnea by means of the Modified Medical Research Council (MMRC) scale [8].

CAT is an 8-item unidimensional measure of individual health status impairment in patients with COPD, of the impact of COPD on a person's life, and how this modifies over time. It contains eight short, simple questions answered by the patient by choosing a score from 0 to 5 for the extent to which the described impairment is deemed true. The CAT score is calculated as the sum of the responded items, ranging from 0 to 40. A higher score indicates a greater impact of symptoms on patient daily activity.

The MMRC dyspnea scale has been in use for many years to evaluate the effect of breathlessness on daily activities, by measuring the perceived respiratory disability, according to the following dyspnea grades:

0 - Breathless with strenuous exercise

1 - Short of breath when hurrying on the level or walking up a slight hill

2 - Walks slower than people of the same age on the level because of breathlessness or stops for breath when walking at own pace on the level

3 - Stops for breath after walking about 100 meters or after a few minutes on the level

4 - Too breathless to leave the house or breathless when dressing or undressing

In the MMRC 0 - 4 grading system a higher score indicates a higher level of dyspnea. All questionnaires were completed by the patients at enrolment, 6- and 12-month follow up visits. during a 12-month observation period.

In addition, socio-demographic variables, smoking habits, medical history at baseline and lung function test results (by means of spirometry), COPD exacerbations, disease severity and medications for COPD, COPD exacerbations and adverse events during follow up were collected. At each visit, each patient filled in a structured interview aimed at assessing his/her awareness of the disease. A comprehensive assessment schedule, mirroring the routine clinical care of most patients, is shown in Table 1.

Size of the study. The sample size was determined based on the feasibility criteria. According to the number of patients managed by the centers involved in this study, the inclusion of about 400 subjects (20 patients/center) with the defined inclusion/exclusion criteria was appropriate for the enrollment period that was initially planned (i.e. 8 months).

Data analysis. Database management and data analysis were performed using the SAS® 9.4 software. Descriptive statistics were used to evaluate socio-demographic and clinical variables at the baseline visit and during the study period. Mean, standard deviation, median, first and third quartiles, minimum and maximum values, proportions (with their respective 95% confidence intervals and standard errors when relevant) were calculated for numerical variables that were analyzed.

The absolute and relative frequency of each variable level was computed for the analyzed categorical variables. Multivariate linear regression analysis was used to evaluate the relationship between demographic or clinical parameters and the level of treatment satisfaction of the patients with COPD enrolled in the study. Linear regression models for repeated measures were used to estimate the β coefficients for the evaluation of the associations between the three TSQM-9 treatment satisfaction domains scores [9] (effectiveness, convenience, and global satisfaction of COPD treatments, each ranging 0-100 points). The following independent factors were evaluated: age and gender, B-IPQ total score, FEV1 % of the predicted, number of annual exacerbations, MMMS-4 score (poor/suboptimal versus optimal), MMRC dyspnea grade, COPD therapeutic regimen. Only patients with data available both at enrollment and at 12-month follow-up visits were included in the regression models. Patients with missing values were not excluded from the analysis, their data were not replaced. Lost to follow up patients were analyzed until their last available visit.

Table 1. Factors associated with treatment satisfaction: Patients' distribution by CAT-score and mMRC-scale changes at 12 months compared to baseline

	N	%
**Extent of CAT score change at 12 months from enrollment**		
CAT decrease of >=2 points	126	41.0
No relevant CAT score change	57	18.6
CAT increase of >=2 points	124	40.4
Evaluable patients at 12 months with available data(*)	307	100.0
**Extent of mMRC score change at 12 months from enrollment**		
mMRC decrease of >=1 point	67	22.0
No relevant mMRC score change	160	52.5
mMRC increase of >=1 point	78	25.6
**Evaluable patients at 12 months with available data(*)**	305	100.0

95% confidence interval limits of calculated means

**Effectiveness score changes and CAT**	Mean	Lower limit	Upper limit
Effectiveness score change in pts with CAT decrease of >=2 points	4.8	1.4	8.2
Effectiveness score change in pts with no relevant CAT change	5.1	1.2	8.9
Effectiveness score change in pts with CAT increase of >=2 points	-0.9	-5.1	3.4

95% confidence interval limits of calculated means

Convenience score changes and CAT	Mean	Lower limit	Upper limit
Convenience score change in pts with CAT decrease of >=2 points	2.6	-0.2	5.3
Convenience score change in pts with no relevant CAT change	0.2	-3.3	3.8
Convenience score change in pts with CAT increase of >=2 points	-4.4	-7.4	-1.4

95% confidence interval limits of calculated means

Global satisfaction score changes and CAT	Mean	Lower limit	Upper limit
Global satisfaction score change in pts with CAT decrease of >=2 points	3.2	-0.1	6.4
Global satisfaction score change in pts with no relevant CAT change	1.1	-3.6	5.9
Global satisfaction score change in pts with CAT increase of >=2 points	-1.0	-4.3	2.4

95% confidence interval limits of calculated means

Effectiveness score changes and mMRC	Mean	Lower limit	Upper limit
Effectiveness score change in pts with mMRC decrease of >=1 points	3.0	-0.7	6.6
Effectiveness score change in pts with no relevant mMRC change	3.6	0.1	7.2
Effectiveness score change in pts with mMRC increase of >=1 points	0.4	-4.1	5.0

95% confidence interval limits of calculated means

Convenience score changes and mMRC	Mean	Lower limit	Upper limit
Convenience score change in pts with mMRC decrease of >=1 points	1.5	-3.0	5.9
Convenience score change in pts with no relevant mMRC change	0.6	-1.7	2.9
Convenience score change in pts with mMRC increase of >=1 points	-4.4	-8.0	-0.8

95% confidence interval limits of calculated means

Global satisfaction score changes and mMRC	Mean	Lower limit	Upper limit
Global satisfaction score change in pts with mMRC decrease of >=1 points	4.1	-0.2	8.4
Global satisfaction score change in pts with no relevant mMRC change	2.0	-0.9	4.9
Global satisfaction score change in pts with mMRC increase of >=1 points	-2.5	-6.6	1.6

References

1. Bettoncelli G, Blasi F, Brusasco V, Centanni S, Corrado A, De Benedetto F, et al. The clinical and integrated management of COPD. An official document of AIMAR (Interdisciplinary Association for Research in Lung Disease), AIPO (Italian Association of Hospital Pulmonologists), SIMER (Italian Society of Respiratory Medicine), SIMG (Italian Society of General Medicine). Multidisciplinary Respiratory Medicine. 2014;9(1):25.

2. Atkinson MJ, Sinha A, Hass SL, Colman SS, Kumar RN, Brod M, et al. Validation of a general measure of treatment satisfaction, the Treatment Satisfaction Questionnaire for Medication (TSQM), using a national panel study of chronic disease. Health Qual Life Outcomes. 2004;2:12.

3. Atkinson MJ, Kumar R, Cappelleri JC, Hass SL. Hierarchical construct validity of the treatment satisfaction questionnaire for medication (TSQM version II) among outpatient pharmacy consumers. Value Health. 2005;8 Suppl 1:S9-S24.

4. Bharmal M, Payne K, Atkinson MJ, Desrosiers MP, Morisky DE, Gemmen E. Validation of an abbreviated Treatment Satisfaction Questionnaire for Medication (TSQM-9) among patients on antihypertensive medications. Health Qual Life Outcomes. 2009;7:36.

5. Broadbent E, Petrie KJ, Main J, Weinman J. The brief illness perception questionnaire. Journal of psychosomatic research. 2006;60(6):631-7.

6. Morisky DE, Green LW, Levine DM. Concurrent and predictive validity of a self-reported measure of medication adherence. Medical care. 1986;24(1):67-74.

7. Jones PW, Harding G, Berry P, Wiklund I, Chen WH, Kline Leidy N. Development and first validation of the COPD Assessment Test. Eur Respir J. 2009;34(3):648-54.

8. Schilling RS, Hughes JP, Dingwall-Fordyce I. Disagreement between observers in an epidemiological study of respiratory disease. British medical journal. 1955;1(4905):65-8.

9. Donner A. Linear regression analysis with repeated measurements. Journal of chronic diseases. 1984;37(6):441-8.
