# Supplementary material for: Satisfaction with chronic obstructive pulmonary disease treatment: results from a multicenter, observational study
Source: Ther Adv Respir Dis. 2019 Nov 24;13:1753466619888128. doi: 10.1177/1753466619888128 (PMC6878607; doi:10.1177/1753466619888128)
Supplement: Reviewer_1_v.1 – Supplemental material for Satisfaction with chronic obstructive pulmonary disease treatment: results from a multicenter, observational study [file Reviewer_1_v.1.pdf]

Reviewer 1 v.1

Comments to the Author

The background (and the title) of the manuscript suggest that it could be a relationship between satisfaction and adherence in COPD patients and this could be the aim of the study, but after that there is no question or any hypothesis, therefore statistical analysis must not use lineal regression which apply only for inferential studies. I suggest to modify the methodology or designe in order to have clear and valid results. I am attaching another study[1] looking at adherence in COPD patients using the same questionnaire, which could be useful.

1. Montes de Oca M, Menezes A, Wehrmeister FC, Lopez Varela MV, Casas A, Ugalde L, et al. (2017) Adherence to inhaled therapies of COPD patients from seven Latin American countries: The LASSYC study. *PLoS ONE* 12(11): e0186777. https://doi.org/10.1371/journal. pone.0186777
