# Supplementary material for: Satisfaction with chronic obstructive pulmonary disease treatment: results from a multicenter, observational study
Source: Ther Adv Respir Dis. 2019 Nov 24;13:1753466619888128. doi: 10.1177/1753466619888128 (PMC6878607; doi:10.1177/1753466619888128)
Supplement: Reviewer_2_v.1 – Supplemental material for Satisfaction with chronic obstructive pulmonary disease treatment: results from a multicenter, observational study [file Reviewer_2_v.1.pdf]

Reviewer 2 v.1 Comments to the Author

The manuscript entitled "SATisfaction and adherence to Chronic Obstructive Pulmonary Disease (COPD) treatment: results from a multicenter, observational study" analyze the results of a national - multicenter, prospective longitudinal study aimed to explore COPD patients' satisfaction to treatment and association with clinical parameters, adherence to treatment and illness perception (assessed by validated questionnaires).

This is an interesting study with useful, relevant and important information in this field, but this manuscript has some limitations and drawbacks both in the design of the study as well as in the interpretation of the results.

The main points of my criticism can be summarized as follows:

• The authors stated that "In summary, no clinically meaningful changes were observed in the overall patients' disease perception, adherence, health status and dyspnea severity over 12 months". The authors report only statistical differences in all reported outcomes and not clinical meaningful differences (e.g percentage of patients with change in MRC scale or CAT changes equal or more of 2 or 3 points).

• The authors stated that "At each visit, data on switching/modification of inhaled treatments and exacerbation events occurred from the previous visit were collected". But neither in the results or in the discussion section, the prospective data on exacerbation frequency were reported or analyzed. What was the proportion of patients with 1 or more exacerbation in the 1 year follow-up period and was there any differences in this population in the parameters tested – mainly satisfaction and adherence to treatment?

• The authors stated that "The results of this analysis revealed that patients' satisfaction is associated mainly with a low perception of the disease". How does this result match with the authors' conclusion that "…a more confident approach of the patient towards the illness promotes the satisfaction with the ongoing treatment"?

• Were there any differences in satisfaction and/or adherence to treatment regarding the inhaler device (MDI vs DPI)? Are these data available for analysis?

• P values are needed to include in Tables 3 to 5.
