# Supplementary material for: Satisfaction with chronic obstructive pulmonary disease treatment: results from a multicenter, observational study
Source: Ther Adv Respir Dis. 2019 Nov 24;13:1753466619888128. doi: 10.1177/1753466619888128 (PMC6878607; doi:10.1177/1753466619888128)
Supplement: Author_Response_1 – Supplemental material for Satisfaction with chronic obstructive pulmonary disease treatment: results from a multicenter, observational study [file Author_Response_1.pdf]

Author Response 1

Responses to Reviewer #1:

We thank the reviewer for her/his helpful comment that we addressed point-by-point. The revised manuscript has been amended accordingly.

Reviewer 1 general Comments:

The background (and the title) of the manuscript suggest that it could be a relationship between satisfaction and adherence in COPD patients and this could be the aim of the study, but after that there is no question or any hypothesis, therefore statistical analysis must not use lineal regression which apply only for inferential studies. I suggest to modify the methodology or designe in order to have clear and valid results. I am attaching another study looking at adherence in COPD patients using the same questionnaire, which could be useful.

Response to Reviewer 1 general comment.

We thank the reviewer for her/his comment that gives us the opportunity to further clarify our study design and aims. This was a real-world, non-inferential study with a descriptive purpose. In fact, in the "Methods" section of the original version of the manuscript, it was stated that "this was a descriptive study, no formal statistical hypotheses were set". As we detailed in the updated manuscript (for the sake of clarity), the primary objective of the study was "to describe patients' satisfaction to COPD medical treatments by means of the TSQM-9 during a 12-month observation period in a real-world setting". However, as exploratory analysis of interest, we also investigated (as specified in the method section) the association between treatment satisfaction and clinical characteristics (including also adherence, disease perception, health status, and dyspnea). The purpose of this analysis was not to obtain a predictive model, but to explore possible associated factors among selected parameters of clinical interest. Therefore, even if no a priori hypotheses were drawn, performing exploratory linear regression analysis is not a methodological flaw, even if results should be interpreted with caution (since the study was not designed to demonstrate a defined relationship). Regarding the statistical approach, the choice of a multivariate linear regression analysis was deemed to be appropriate to investigate factors possibly associated to treatment satisfaction. Moreover, with respect to the methods used in the provided article [Montes de Oca M et al, 2017], the calculation of Cohen's kappa index was not applicable (because the evaluation of agreement between two scales was not within the scope of the SAT study), and the Poisson regression model was not suitable (since the dependent variables in our models were numerical, and not a count of events). Following reviewer's comment, we modified both the title of the manuscript and the introduction section accordingly, in order to highlight that the SAT study was primarily focused in describing long-term treatment satisfaction from the patient's perspective. We now also report in the "Discussion" section that inferential studies are needed to confirm our preliminary findings regarding the putative factors associated to COPD treatment satisfaction.

Responses to Reviewer #2:

We thank the reviewer for her/his helpful comment that we addressed point-by-point. The revised manuscript has been amended accordingly.

Reviewer 2 general Comments:

The manuscript entitled "SATisfaction and adherence to Chronic Obstructive Pulmonary Disease (COPD) treatment: results from a multicenter, observational study" analyze the results of a national - multicenter, prospective longitudinal study aimed to explore COPD patients' satisfaction to treatment and association with clinical parameters, adherence to treatment and illness perception (assessed by validated questionnaires). This is an interesting study with useful, relevant and important information in this field, but this manuscript has some limitations and drawbacks both in the design of the study as well as in the interpretation of the results.

Response to reviewer 2 general comment

we thank the reviewer for the positive consideration to our study. As requested, the helpful comments provided by the reviewer have been addressed point-by-point and the manuscript amended accordingly.

Reviewer 2 comment 1 (R2C1).

The authors stated that "In summary, no clinically meaningful changes were observed in the overall patients' disease perception, adherence, health status and dyspnea severity over 12 months". The authors report only statistical differences in all reported outcomes and not clinical meaningful differences (e.g percentage of patients with change in MRC scale or CAT changes equal or more of 2 or 3 points).

Response to reviewer 2 comment 1 (RR2C2).

we thank the author for her/his comment that gives us the possibility to further clarify the results of our study. For these reasons, and following reviewer comments, in the revised version of the manuscript we avoid using the terminology "clinically meaningful" when referring to changes in the TSQM-9, B-IPQ and MMAS-4 questionnaires. We now also provide a new analysis reporting the percentage of patients whose changes in CAT score and/or mMMC scale reached the MCID at 12 months compared to baseline. Overall, we found that a significant proportion of patients reached the MCID in the CAT score (+/- 2 points) and in the mMRC scale (+/- 1 point) after the 12-month observation period as compared to baseline. In patients with a decreased in CAT score (reflecting the improvement of the impact of COPD on patient's life) we found and increase of all the domains of the TSQM-9 questionaire (meaning an improvement of patients' satisfaction to treatment). Similarly, we found and increase of all the domains of the TSQM-9 questionaire in COPD patients with an increase of at least 1 point in the mMRC scale at the end of the study period compared to baseline. These data are now reported in the revised version of the manuscript.

R2C2.

The authors stated that "At each visit, data on switching/modification of inhaled treatments and exacerbation events occurred from the previous visit were collected". But neither in the results or in the discussion section, the prospective data on exacerbation frequency were reported or analyzed. What was the proportion of patients with 1 or more exacerbation in the 1 year follow-up period and

was there any differences in this population in the parameters tested – mainly satisfaction and adherence to treatment?

RR2C2.

We thank the reviewer for rising this relevant point that gives us the opportunity to provide further results and analysis to our study. The definition of exacerbation and of the severy of the exacerbation is reported in Study procedures, variables, and outcomes of the revised version of the manuscript. In particular we now report that the average number of exacerbations per patient during observation period was 0.3 event/patient (SD=0.6) and that 99 patients (24.7%) had at least one new exacerbation during the 12-month observation period. Overall, no statistically significant associations were found between patients' satisfaction (any of the TSQM-9 domains) and the frequency of exacerbations. Very few events of hospitalization occurred during the follow up period (only 9 events corresponding to 2.2 events/100 patients). A weak statistically significant negative correlation between the convenience item of the patients' satisfaction score (exploring satisfaction to treatment regimen, dosing complexity and frequency) and the total number of hospitalizations (ρ=-0.13, P-value=0.02, N=304) was found. This finding suggests a greater satisfaction in subjects who had less COPD-related hospitalizations. However, it should be noted that the magnitude of these correlations is very limited, meaning that they may not actually be clinically relevant. Therefore, these findings should be interpreted with caution given the weak nature of the associations observed. This is now discussed also in the revised version of the discussion.

As reported in the method section (see also response to reviewer 1) the primary objective of the study was to describe patients' satisfaction to COPD medical treatments. Furthermore, as exploratory analysis of interest, we investigated also the association between treatment satisfaction and clinical characteristics (including adherence). For these reason the correlation between aderenche to treatment and exacerbation frequency has not been primarily explored. Happy to provide it if considered essential.

R2C3

The authors stated that "The results of this analysis revealed that patients' satisfaction is associated mainly with a low perception of the disease". How does this result match with the authors' conclusion that "…a more confident approach of the patient towards the illness promotes the satisfaction with the ongoing treatment"?

RR2C3

We thank the author for the comment, and we apologize for the misleading/not clear sentence provided in the original version of the manuscript. The reviewer is right: we found a negative association between satisfaction and disease perception. The misleading sentence has been removed and the paragraph in the discussion section has been rephrased.

R2C4

Were there any differences in satisfaction and/or adherence to treatment regarding the inhaler device (MDI vs DPI)? Are these data available for analysis?

RR2C4

We thank the reviewer for the comment. In the manuscript we report that, similar levels of satisfaction to treatments were found during the 12-mounth follow up period between patients who switched and who did not switch the pharmacological inhaled regimens. Whether the pharmacological change was related to the modification of the compounds and/or in the device has not been sistematicalluy made available. Thus, the interesting analysis suggested by the reviewer cannot be formally performed and it deserves a specific study design.

R2C5

P values are needed to include in Tables 3 to 5.

RR2C5

For Table 3, statistical testing was not foreseen coherently with the descriptive aim of the analysis. In fact, as specified in the "Methods" section of the manuscript, "this was a descriptive study, no formal statistical hypotheses were set". The provided 95% confidence interval limits inform on the estimate precision of the mean scores changes over time; at the same time, if the value 0.0 falls within 95% CI limits, it can be deduced that the calculated difference would not reach statistical significance (with $\alpha=0.05$). However, even if statistical testing were performed and significant changes over time were discovered, we would not claim the reported results to be "relevant changes" from a clinical point of view: in fact, the observed overall extent of variation in B-IPQ, MMAS-4, CAT, and mMRC scores was deemed to be limited. As an example, the 12-month intra-patient change in B-IPQ score accounted for a mean increase of 1.3 [95% CI: 0.2; 2.3] points; however, if compared to the theoretical range of possible scale values (max value: 80 points), it might be pointed out that this increment is negligible (even if no validated cut-off or MCID scores are available). However, since the value 0 is comprised between the 95% CI limits, if a p-value was computed for this score change, this would result in a statistically significant change–despite being a numerically small change. In order to avoid the readers to draw misleading conclusions, and according to the non-inferential primary aim of the study, we believe that p-value calculation is not indicated for this analysis.

- Table 4 already contains p-values, which were necessary to explore which factors were possibly associated with treatment satisfaction.

- Table 5 was provided only for informative purposes, but the analysis was not aimed to identify which treatments the patients were most satisfied to. There were no pre-specified hypotheses, and comparisons between type of treatment classes were not of interest. Moreover, the group size were not homogeneous, and many patients groups were definitely too small for a robust comparative analysis (which might be affected by risk of false positive results).
